# Symbiotic revolutions at the interface of genomics and microbiology

**DOI:** 10.1371/journal.pbio.3002581

**Published:** 2024-04-09

**Authors:** John M. Archibald

**Affiliations:** 1 Department of Biochemistry & Molecular Biology, Dalhousie University, Halifax, Canada; 2 Institute for Comparative Genomics, Dalhousie University, Halifax, Canada; University of Texas Austin, UNITED STATES

## Abstract

Symbiosis is an old idea with a contentious history. New genomic technologies and research paradigms are fueling a shift in some of its central tenets; this Perspective argues that we need to be humble and open-minded about what the data are telling us.

## Teaser

Symbiosis is an old idea with a contentious history. New genomic technologies and research paradigms are fueling a shift in some of its central tenets; we need to be humble and open-minded about what the data are telling us.

In the 1920s, an American professor named Ivan Wallin (1883–1969) published a string of articles and a book about mitochondria. Wallin’s day job was teaching anatomy to medical students at the University of Colorado, but he was also fascinated by cell biology. Over the course of a decade, Wallin forged the concept of “symbionticism,” whereby speciation in animals and plants is triggered by the acquisition of bacterial symbionts. Wallin believed that mitochondria were such symbionts and had evidence to prove it—his publications included *camera lucida* drawings of “mitochondria” growing happily on agar plates! Wallin was not the first to claim that mitochondria could be cultured. The Frenchman Paul Portier (1866–1962) made similar noises in his 1918 book *Les Symbiote*s. With the benefit of hindsight, of course, we know that they could not possibly have cultivated mitochondria [1]. Mitochondria are not bacteria; they evolved from bacteria around 2 billion years ago and are permanent fixtures of the eukaryotic cytoplasm—the “mitochondria” observed in their experiments can only have been bacterial contaminants. But digesting old literature can be fun and instructive. How do the controversial ideas of Portier and Wallin resonate in present-day symbiosis research? One hundred years on, what has changed? Everything and nothing.

Let us start with the obvious: genomics is now “a thing.” First genes and then entire genomes were sequenced, many hundreds of thousands of them from across the tree of life. We have learned that mitochondrial genomes, like those of plastids, encode only a small fraction of the 1,000+ proteins required for organelle function; most such proteins are encoded in the nucleus and imported posttranslationally. We have learned that endosymbiotic gene transfer (EGT) had an important role in the evolution of mitochondria and plastids from alphaproteobacteria and cyanobacteria, respectively, and that EGT still occurs today. Wallin was wrong to insist that symbiosis and speciation went hand in hand, but he was onto something when he proposed that gene transfer between closely associated organisms might be important [1]. Another recurring theme is metabolic complementarity, which today can be predicted using comparative genomics and tested experimentally. Consider lichens, the classic fungal–algal (or cyanobacterial) symbiosis taught in high school. Here, genomics has confirmed the basics but also revealed that the traditional two-partner lichen model based on nutrient exchange and protection is overly simplistic. Multiple fungi and a zoo of bacteria can in fact be found in lichens, and the metabolic versatility of the fungi is much greater than previously assumed [2]. As in many other areas of symbiosis research, comparative genomics has been a hypothesis-generating machine for today’s lichenologists.

Lest we get too confident in the robustness of our data and interpretations, recall the controversy surrounding horizontal gene transfer (HGT) in tardigrades (water bears), and claims that foreign gene acquisition underpins their exceptional tolerance to stress. Fully one-sixth of the genes in the tardigrade genome were initially proposed to be HGTs; many (most?) of these now appear to be the result of contamination [3]. Another cautionary tale is the decade-long debate about whether alga-to-animal gene transfer supports kleptoplasty (“plastid stealing”) in sea slugs (the answer is “no” [4]). Such examples remind us that biology is messy, and the art of genome assembly is a work in progress. Even with the advent of long-read sequencing, most genome sequencing projects are, bioinformatically speaking, best treated as metagenomic journeys into the unknown.

What did Wallin and Portier know? Beyond aphids, slugs, and lichens, they knew about symbioses involving sponges, corals, *Hydra*, and bobtail squid, and that protists often have algal or cyanobacterial endosymbionts. From this, they sought to develop general principles to explain how and why cells and organisms interact in nature. The field of microbiology was, at the time, medically oriented and dominated by the following sentiment: microbes are bad. When bacteria, protists, and fungi were found near or within animal and plant cells, the default lens was one of pathology. Portier and Wallin saw things differently.

Which brings us to microbiomics, an exploratory, tech-driven field in which no environment is off limits: if biomass can be collected and DNA extracted, metagenomes can be sequenced. We can ask “who is there?” and “what are they doing?” without a microscope or the need to culture. The results have been breathtaking. In 2015, a new superphylum of Archaea, the Asgardarchaeota, was discovered from deep-sea sediments, and appears to represent the archaeal branch from which the host component of the eukaryotic cell evolved [5]. A 2016 analysis of metabarcode data revealed that diplonemids, a protist lineage for which only a handful of species have ever been described, are in fact among the most abundant predators in the sea [6]. And in 2023, a new lineage of marine viruses—mirusviruses (*Mirus* is Latin for strange)—was identified using metagenomics; these viruses appear to have a broad host range within protists [7].

Our understanding of microbial diversity is changing and so is the language we use to describe it. Recent years have seen fruitful dialog between organelle researchers, those focused on animal and plant symbioses, and human microbiome researchers—all are leveraging the awesome power of “omics” but are inclined to define and interpret their data in different conceptual frameworks. Consequently, new terms are cropping up and existing ones have become less precise. Consider “microbiome.” It is now commonplace to refer to the ocean microbiome and the soil microbiome, even the microbiome of glaciers. At the other end of the spectrum, we now speak of the microbiomes of single-celled eukaryotes. Somewhere in between are the microbiomes of plant roots, honey bees, and humans. Where does a microbiome end and a symbiotic consortium begin? What matters most, a microbiome’s ecological context, its functional repertoire, its taxonomic composition, or the extent to which it is heritable across time and space? There are no easy answers, and introduction of new(er) terms such as “holobiont” and “hologenome” have brought age-old questions about individuality and levels of selection into the debate [8,9].

The history of science teaches us that we should be open-minded and recognize the value of different perspectives. Debate is healthy. New synthetic frameworks will emerge. And at the interface of symbiosis and microbiomics, the perfect complement to holism is reductionist methodology. The goal here is to combine omics tools with microscopy and genetics to study symbiosis in the lab to systematically “turn the knobs” so that we can isolate and understand the variables that determine how and why organisms live together. On this front, the future is bright. With their Symbiosis in Aquatic Systems initiative, the Gordon and Betty Moore Foundation is investing heavily in genetic tools development for marine protists [10], and in doing so, paving the way for controlled experimentation on symbioses from diverse branches of life’s tree.

At the present time, it is safe to say that symbioses are not the utopian relationships we once assumed them to be. Careful research has shown that in the social soil amoeba *Dictyostelium*, “farmed” *Burkholderia* bacteria can have mutualistic or harmful effects on their hosts depending on the environmental conditions [11]. The long-studied facultative symbiosis between the ciliate *Paramecium bursaria* and the green alga *Chlorella* has recently been described as a case of “controlled exploitation,” one in which fluctuations in the amount of food and sunlight can tip the cost–benefit scale one way or the other [12]. In the 1860s, the fungal–algal duality of lichens was originally cast as “master–slave,” then as a mutualism; now it is a “multiplayer marketplace of rewards and penalties” [2]. In nature’s game of host–symbiont interactions, the answer to the question of who benefits and how is increasingly “it depends” [13].

The Ivan Wallin Fan Club has few members, but it did include the undisputed champion of endosymbiotic theory, Lynn Margulis (1938–2011). In 1993, Margulis published Wallin’s final manuscript [14], discovered among his papers in Denver, which had been submitted for publication in 1969, the year he died and more than 40 years after he had stopped doing research. Scientists had recently used electron microscopy to demonstrate the presence of DNA in mitochondria and plastids, and Wallin seized the opportunity to make a final plea for the bacterial essence of mitochondria. He noted that “some of these investigators have indicated that mitochondria have their origins in bacteria which have invaded the cell” [14]. He also stood by his earlier claims to have cultured mitochondria. The manuscript was rejected by *Science* “without comment.”
